# Association between CYP2A13 polymorphisms and lung cancer: A protocol for systematic review and meta-analysis

**DOI:** 10.1097/MD.0000000000023289

**Published:** 2020-12-11

**Authors:** Long Ma, Gang Jin, Yi Yang, Yao Pang, Wenhao Wang, Hongyi Zhang, Jiawei Liu, Peng Wu, Zequan Wang, Kui Wang, Ruitong Chang, Jialong Li, Zijiang Zhu

**Affiliations:** a Department of Clinical Medicine, Gansu University of Traditional Chinese Medicine; b Department of Thoracic Surgery, Gansu Provincial Hospital, Lanzhou, China.

**Keywords:** cytochrome P450 2A13, lung cancer, meta-analysis, polymorphism

## Abstract

**Background:**

Recently, lung cancer has become the most common cause of cancer-related death, several studies indicate that the cytochrome P450 2A13 (CYP2A13) polymorphisms may be correlated with lung cancer susceptibility, but the results have been inconsistent and inconclusive. Therefore, the aim of this meta-analysis is to provide a precise conclusion on the potential association between CYP2A13 polymorphisms and the risk of lung cancer based on case-control studies.

**Methods:**

The PubMed, Embase, Cochrane Library, Web of Science, and China National Knowledge Infrastructure (CNKI) databases will be searched for case-control studies published up to September 2020. Odds ratio (OR) and 95% confidence interval (95% CI) were used to determine the effects of the CYP2A13 polymorphism on lung cancer risk, respectively.

**Results:**

The results of this meta-analysis will be submitted to a peer-reviewed journal for publication.

**Conclusion:**

This meta-analysis will summarize the association between CYP2A13 polymorphisms and the risk of lung cancer.

**INPLASY registration number:**

INPLASY202090102

## 1 Introduction

In recent years, with changes in the environment, the incidence and mortality of lung cancer have been increasing rapidly.^[1,2]^ Lung cancer is a public health problem that seriously affects human health, the average fatality rate of male lung cancer patients and female lung cancer patients worldwide is 27% and 26%, respectively, ranking first in the mortality rate of malignant tumors.^[3–5]^ The latest cancer statistics report in 2018 found that the number of cancer cases in China in 2014 reached >3.8 million, of which lung cancer cases accounted for 11.6% of the proportion, which brought a serious burden to the family and society.^[6–8]^ Under similar environmental factors, there are significant differences in the risk of lung cancer among different individuals. This phenomenon indicates that the occurrence of lung cancer is also related to individual susceptibility.^[9]^

CYP2A13 is mainly expressed in the respiratory system, with the highest expression in the olfactory mucosa, followed by lungs and trachea.^[10,11]^ In lung tissue, CYP2A13 is mainly located in bronchial epithelial cells.^[10]^ Studies have shown that smoking is an important risk factor for lung cancer.^[12]^ Cigarette smoke containing a lot of chemicals is inhaled through the respiratory system, CYP2A13 metabolizes the nicotine in cigarette smoke and the pro-carcinogen N-nitroso (4-(methyl-nitrosamine)-1-(3-pyridyl)-1-butanone, NNK), which forms the active metabolites can cause damage to the respiratory system.^[13–18]^ CYP2A13 can efficiently metabolize and activate aflatoxin B1, generate a variety of metabolites including epoxide, and then combine with DNA, RNA, protein, and other biological macromolecules in the body to form DNA, RNA, and protein adducts to cause cytotoxicity, Inflammation, DNA damage, and other toxic effects, which may cause cell malignant transformation and cause body tumors.^[19–25]^ At present, there is no consistent conclusion on the genetic polymorphism of CYP2A13 and lung cancer susceptibility. Therefore, in this study, the meta-analysis method was used to systematically evaluate the relationship between CYP2A13 gene polymorphism and lung cancer susceptibility, in order to provide a basis for exploring clinical drug targets and cancer prevention.

## 2 Methods

### 2.1 Search strategy

We will search the PubMed, Cochrane Library, Web of Science, CNKI databases, and the time period for the reference searches was from the first available article to September 2020. The following key words were used for the searches: “Cytochrome P450 2A13,” “CYP2A13,” “lung cancer,” “lung carcinoma,” “lung tumor,” “polymorphism,” “polymorphisms,” “variant,” “variants.”

### 2.2 Inclusion criteria

(1) Using case-control study method to assess the relationship of CYP2A13 polymorphisms with lung cancer risk.
(2) The size of the sample, odds ratios (ORs), and their 95% confidence intervals (CIs) were provided.
(3) In the case of multiple publications from the same study group, the most complete and recent results will be used.

### 2.3 Exclusion criteria

(1) Multiple publications reporting the same finding.
(2) Abstracts, reviews, comments, and animal studies.
(3) Lack of sufficient data.

### 2.4 Data extraction

Data will be carefully extracted from all selected articles by 2 of the authors, independently. When discrepancies appear, another investigator will be recruited to assess the data. The information extract from the studies included the first author's name, the year of publication, the country of the study, the ethnicity of the subjects, the diagnostic method, the number of cases in the lung cancer group and the control group, and the numbers of each genotype identified.

### 2.5 Study quality assessment

The quality of the included studies will be independently assessed by the Newcastle-Ottawa Quality Assessment Scale (NOS).^[26]^ The evaluation project consisted of 8 parts, and except for the fifth evaluation criterion of 2 points, the scores of the other items are all 1 point. The total scores of the NOS range from 0 points to 9 points. If the total score of the 8 items is ≥7 points, the quality of studies is considered reliable.

### 2.6 Statistical analysis

The statistical analysis will be performed by using Review Manager 5.3. Pooled odds ratios and 95% confidence intervals^[27]^ will be applied to estimate the strength of associations between the CBS gene polymorphisms and lung cancer risk. The heterogeneity between the included studies will be judged on the basis of *P* and *I*^2^ values. Values of *P* > .10 and *I*^2^ < 50% indicate that the fixed effect model could be used in this meta-analysis. However, values of *P* < .10 and *I*^2^ > 50% indicate the existence of significant heterogeneity.^[28]^ We will identify the source of heterogeneity and perform further analysis, and the random effects model will be used after excluding the effects of significant clinical heterogeneity.

### 2.7 Subgroup analysis

To further study the effect of sample size, diagnosis methods and ethnicity on heterogeneity, subgroup analysis will be performed.

### 2.8 Assessment of publication bias

The funnel plot and Egger test will be conducted to detect publication bias if the number of studies >10.

### 2.9 Sensitivity analysis

We will perform a sensitivity analysis to verify the robustness of the study results. This will be achieved by assessing the impact of the sample size, high risk of bias, missing data, and selected models. Following the analyses, if the quality of a study is judged to be low, it will be removed to ensure the robustness of the results.

### 2.10 Ethics and dissemination

Ethical approvals patient consent are not required because this is a meta-analysis based on published trials. The finding of this project will provide a general review and evidence of the association between CYP2A13 polymorphisms and the risk of lung cancer. The results will be submitted to a peer-reviewed journal for publication. We hope that finding will help clinicians can carry out early screening, early diagnosis, and early treatment of lung cancer patients.

## 3 Discussion

This systematic review will be the first to assess the association between CYP2A13 polymorphisms and the risk of lung cancer. The review contains 5 sections: search strategy, study inclusion and exclusion, data extraction, statistical analysis, subgroup analysis. The results will be submitted to a peer-reviewed journal for publication. We hope that finding will help clinicians can carry out early screening, early diagnosis, and early treatment of lung cancer patients.

## Author contributions

**Conceptualization:** Long Ma, Gang Jin, Yi Yang, Zijiang Zhu.

**Data curation:** Long Ma, Gang Jin, Yi Yang, Yao Pang, Wenhao Wang, Hongyi Zhang, Jiawei Liu, Peng Wu, Zequan Wang, Kui Wang, Ruitong Chang, Jialong Li.

**Formal analysis:** Long Ma, Gang Jin, Yi Yang, Yao Pang, Wenhao Wang, Hongyi Zhang, Jiawei Liu, Peng Wu, Zequan Wang, Kui Wang, Ruitong Chang, Jialong Li.

**Funding acquisition:** Long Ma, Gang Jin, Yi Yang, Yao Pang, Wenhao Wang, Zijiang Zhu.

**Investigation:** Long Ma, Gang Jin, Yi Yang, Yao Pang, Wenhao Wang, Hongyi Zhang, Jiawei Liu, Peng Wu, Zequan Wang, Kui Wang, Ruitong Chang, Jialong Li.

**Methodology:** Long Ma, Gang Jin, Yi Yang, Hongyi Zhang, Jiawei Liu, Peng Wu, Zequan Wang, Kui Wang, Ruitong Chang, Jialong Li, Zijiang Zhu.

**Project administration:** Long Ma, Gang Jin, Yi Yang, Yao Pang, Wenhao Wang, Hongyi Zhang, Jiawei Liu, Peng Wu, Zequan Wang, Kui Wang, Ruitong Chang, Jialong Li.

**Resources:** Long Ma, Gang Jin, Yi Yang, Yao Pang, Wenhao Wang, Hongyi Zhang, Jiawei Liu, Peng Wu, Zequan Wang, Kui Wang, Ruitong Chang, Jialong Li.

**Software:** Long Ma, Gang Jin, Yi Yang, Wenhao Wang, Jiawei Liu, Zequan Wang, Zijiang Zhu.

**Supervision:** Long Ma, Gang Jin, Yi Yang, Zijiang Zhu.

**Validation:** Long Ma, Gang Jin, Yi Yang, Yao Pang, Wenhao Wang, Hongyi Zhang, Jiawei Liu, Peng Wu, Zequan Wang, Kui Wang, Ruitong Chang, Jialong Li.

**Visualization:** Long Ma, Gang Jin, Yi Yang, Yao Pang, Wenhao Wang, Hongyi Zhang, Jiawei Liu, Peng Wu, Zequan Wang, Kui Wang, Ruitong Chang, Jialong Li.

**Writing – original draft:** Long Ma, Gang Jin, Yi Yang, Zijiang Zhu.

**Writing – review & editing:** Long Ma, Gang Jin, Yi Yang, Zijiang Zhu.
